# Root microbes can improve plant tolerance to insect damage: A systematic review and meta‐analysis

**DOI:** 10.1002/ecy.4502

**Published:** 2025-01-21

**Authors:** Emily Tronson, Laramy Enders

**Affiliations:** ^1^ Entomology Department Purdue University West Lafayette Indiana USA

**Keywords:** insect herbivory, meta‐analysis, microbe, microbiome, plant defenses, plant–insect interactions, tolerance

## Abstract

To limit damage from insect herbivores, plants rely on a blend of defensive mechanisms that includes partnerships with beneficial microbes, particularly those inhabiting roots. While ample evidence exists for microbially mediated resistance responses that directly target insects through changing phytotoxin and volatile profiles, we know surprisingly little about the microbial underpinnings of plant tolerance. Tolerance defenses counteract insect damage via shifts in plant physiology that reallocate resources to fuel compensatory growth, improve photosynthetic efficiency, and reduce oxidative stress. Despite being a powerful mitigator of insect damage, tolerance remains an understudied realm of plant defenses. Here, we propose a novel conceptual framework that can be broadly applied across study systems to characterize microbial impacts on expression of tolerance defenses. We conducted a systematic review of studies quantifying the impact of rhizosphere microbial inoculants on plant tolerance to herbivory based on several measures—biomass, oxidative stress mitigation, or photosynthesis. We identified 40 studies, most of which focused on chewing herbivores (*n* = 31) and plant growth parameters (e.g., biomass). Next, we performed a meta‐analysis investigating the impact of microbial inoculants on plant tolerance to herbivory, which was measured via differences in plant biomass, and compared across key microbe, insect, and plant traits. Thirty‐five papers comprising 113 observations were included in this meta‐analysis, with effect sizes (Hedges' *d*) ranging from −4.67 (susceptible) to 18.38 (overcompensation). Overall, microbial inoculants significantly reduce the cost of herbivory via plant growth promotion, with overcompensation and compensation comprising 25% of observations of microbial‐mediated tolerance. The grand mean effect size 0.99 [0.49; 1.49] indicates that the addition of a microbial inoculant increased plant biomass by ~1 SD under herbivore stress, thus improving tolerance. This effect was influenced most by microbial attributes, including functional guild and total soil community diversity. Overall, results highlight the need for additional investigation of microbially mediated plant tolerance, particularly in sap‐feeding insects and across a more comprehensive range of tolerance mechanisms. Such attention would round out our current understanding of anti‐herbivore plant defenses, offer insight into the underlying mechanisms that promote resilience to insect stress, and inform the application of microbial biotechnology to support sustainable agricultural practices.

## INTRODUCTION

When facing herbivore attack, plants do not react alone. The expression of plant defenses shapes and is shaped by a plant's root microbiome (Friman et al., 2021; Rasmann et al., 2017). This community of fungi, bacteria, archaea, protists, and other microorganisms can mediate plant defense expression most notably by triggering induced systemic resistance, in which a plant is primed to respond more effectively to insect attack (Pieterse et al., 2014). Positive microbial‐mediated effects on resistance pathways, such as secondary metabolite production (Contreras‐Cornejo et al., 2016; Matilla & Krell, 2018; Shoresh et al., 2010), reduction in insect growth (Valenzuela‐Soto et al., 2010), and plant volatile production to attract herbivore natural enemies (Guerrieri et al., 2004; Hempel et al., 2009) have also been described. However, compared to plant resistance mechanisms, microbial involvement in tolerance is relatively understudied (Peterson et al., 2017). This side of a plant's defensive repertoire seeks to minimize fitness losses to herbivory, often through modifications to plant architecture, resource allocation (Stowe et al., 2000; Strauss & Agrawal, 1999), photosynthesis, and oxidative stress responses (Koch et al., 2016). By targeting plant physiology rather than insect biology and behavior, tolerance mechanisms avoid exerting selective pressures that can trigger escalating insect virulence and potentially render plant resistance mechanisms ineffective over time (Rausher, 2001). Instead, tolerance is considered to provide an evolutionarily sustainable route through which to counteract insect damage (Espinosa & Fornoni, 2006; Pedigo & Higley, 1992; Rausher, 2001; but see Fornoni, 2011).

Evidence for microbial contributions to tolerance responses exists but remains scattered in the literature and is rarely discussed within the broader framework of plant defenses. The lack of an integrated discussion around microbial‐mediated tolerance may arise from difficulties surrounding studying tolerance itself. For one, the genetic basis of plant tolerance is likely more complex (Stowe et al., 2000) and is less understood than that of resistance (Peterson et al., 2017; Smith & Clement, 2012). Tolerance is a complex phenomenon that results from the integrated expression of traits before and after insect damage occurs, such as resource allocation patterns, plant architecture, and photosynthetic activity, which ultimately impact whole‐plant fitness (e.g., reproduction, survival). In addition, quantifying or imposing standard intensities of herbivore damage is feasible for aboveground chewers, but much less attainable for other feeding guilds (Stowe et al., 2000). Furthermore, while tolerance is ideally measured using plant fitness or yield, studies often use different phenotyping approaches to approximate fitness (e.g., growth rates, floral traits, biomass accumulation) that can vary widely across insect feeding guilds (Enders & Begcy, 2021; Koch et al., 2016; Strauss & Agrawal, 1999; Tao et al., 2016; Tiffin & Inouye, 2000). Consequently, we lack a holistic understanding of microbial involvement in plant tolerance defenses.

Mycorrhizal fungi were among the first root mutualists hypothesized to be involved in “modification of tolerance” via stimulating plant growth and altering nutrient storage patterns (Bennett et al., 2006; Bennett & Bever, 2007). More recent studies involving root‐associated fungi and bacteria have begun to identify potential mechanisms behind tolerance‐promoting mutualisms. In plants exposed to herbivory, root microbes can counteract negative impacts on growth and reproduction (Bernaola & Stout, 2021; Contreras‐Cornejo et al., 2021; Herman et al., 2008), mediate resource reallocation (Frew et al., 2020), and reduce oxidative stress (Selvaraj et al., 2020). While studies thus far show promise for microbial involvement in plant tolerance, positive, neutral, and negative effects of root microbes on plant tolerance to herbivory have all been observed (Bennett & Bever, 2007; Kula et al., 2004). These cases span a wide range of insect herbivores, crop and wild plants, and beneficial fungal and bacterial taxa. As a result, gaps remain in our understanding of how factors such as insect feeding guild (sap‐sucking vs. defoliating), domestication history, and rhizosphere diversity influence microbially mediated tolerance mechanisms. For example, though domestication has altered crop plant root architecture, exudation (Iannucci et al., 2017), and ability to recruit and benefit from interactions with root microbes (Jaiswal et al., 2020; Valente et al., 2020), it is unclear whether these changes have impacted how root microbes influence tolerance.

**FIGURE 1:**
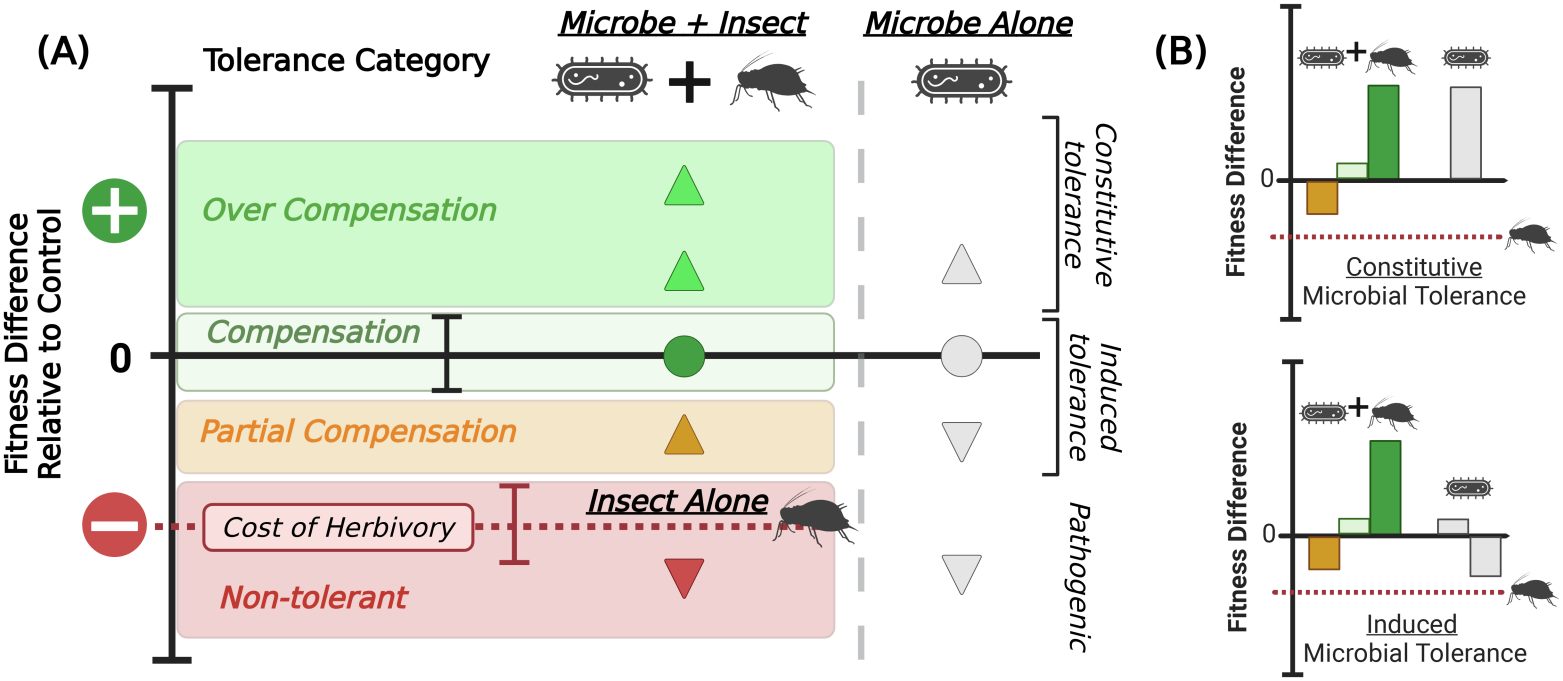
Framework for characterizing microbial involvement in tolerance using plant fitness comparisons across four groups: Control (no insect, no microbe), insect alone (−microbe/+insect), microbe alone (+microbe/−insect), microbe and insect combined (+microbe/+insect). (A) Fitness comparisons relative to the control group are used to identify a “cost to herbivory” in the infested host plant group and microbial‐mediated tolerance responses in the infested holobiont group: *Partial compensation*, *compensation*, and *over‐compensation*. Non‐tolerant cases are also shown, where adding a microbial inoculant does not counteract the cost of herbivory or further reduces fitness of infested host plants. Error bars indicate potential fitness variation across samples. (B) Scenarios for identifying constitutive (microbe alone > control) or induced (microbe alone ≤ control) tolerance responses. Figure generated using BioRender.

To address these gaps, we conducted a systematic review and meta‐analysis of evidence for microbial involvement in tolerance responses. Our goals were to (1) provide a synthesis of existing research on root microbial involvement in tolerance mechanisms and (2) examine how microbial‐mediated tolerance varies with key attributes of insects (e.g., feeding guild), plants (e.g., cultivation status), and root microbial communities (e.g., species richness). However, we currently lack a unified framework for identifying and quantifying the role microbes play in the expression of plant tolerance, particularly one that facilitates comparisons across herbivore type (e.g., feeding guilds, coevolutionary histories with a host plant) and microbial dimensions (e.g., functions, community diversity). We therefore propose a conceptual framework for categorizing microbe‐mediated tolerance to herbivory (Figure 1) that is based on the broad definition of tolerance—a reduction in the fitness loss due to herbivory (Stowe et al., 2000; Strauss & Agrawal, 1999). At its simplest level, tolerance can be estimated as the difference between insect‐infested and uninfested plant fitness, which can then be evaluated in the presence or absence of potential microbial influencers. This framework thus differentiates between host plant and microbial contributions to the expression of tolerance by the holobiont (host + microbiome). Microbial involvement in the expression of tolerance can therefore range from *no effect* when microbial additions neither increase nor decrease fitness losses to herbivory; *susceptibility* or *non‐tolerance* when microbes increase fitness losses to herbivory; *partial compensation* when microbes reduce fitness losses, but not enough to match fitness levels of uninfested host plant controls; *compensation* when microbes eliminate fitness losses to herbivory, such that infested holobiont and uninfested host plant fitness is equal; and *overcompensation*, in which microbes contribute to increasing infested holobiont fitness beyond that of uninfested host plant fitness (Figure 1A). In addition, microbial‐mediated effects on tolerance can be further characterized as *constitutive* when impacts on plant fitness are observed in the holobiont regardless of herbivory (i.e., both “microbe + insect” and “microbe alone”; Figure 1B top panel), or *induced* when fitness effects are seen only in the infested holobiont (i.e., not “microbe alone”; Figure 1B bottom panel). Under this framework, all categories of microbial‐mediated tolerance (overcompensation, partial compensation, compensation) and susceptibility can be either induced or constitutively expressed. The advantages of this proposed framework are broad applicability to (1) multiple fitness components or physiological measures (e.g., yield, biomass, photosynthesis, stress response), (2) individual‐ and community‐level approaches (single or multiple insects/microbes), and (3) a range of experimental conditions (e.g., laboratory and field).

## METHODS

### Literature survey and study eligibility criteria

In order to summarize the state of the literature evaluating microbial involvement in plant tolerance to insect herbivory, we conducted a Web of Science search using three search terms capturing plant, insect, and microbe components: (1) toleran* OR compensat* OR yield; (2) herbivo*; and (3) microb* OR bacteri* OR fung* OR mycorrhiza*. This search was first conducted in July 2021, returning 709 results, and a second search was conducted in January 2023 with identical search terms, yielding an additional 144 results. In March 2023, a supplementary search was performed with the keywords (1) photosynthe* OR reactive oxygen species OR oxidative stress, (2) whitefly OR aphid OR *hopper, and (3) microb* OR bacteri* OR fung* OR mycorrhiza*. This supplementary search was conducted to better survey papers that considered components of tolerance outside of biomass compensation and returned 293 papers.

We next identified papers from our primary literature that fit the following criteria: (1) examined interactions between at least one insect herbivore, one root microbe, and one plant; (2) considered herbivore damage that was real rather than simulated (i.e., mechanical damage); or mechanical damage supplemented with insect regurgitant; (3) applied microbial inoculants to plant roots or soil (i.e., excluded cases of strictly shoot inoculation); (4) focused on the root microbial community (i.e., not phyllosphere/aerial plant tissues); (5) considered at least one measure of plant tolerance (e.g., yield, plant biomass, oxidative stress mitigation, or photosynthesis); (6) included no‐microbe control treatments with which to compare treatments that added one or more microbes; and (7) considered wild‐type varieties/cultivars, rather than mutant genotypes. The resultant 40 papers that fit these criteria are summarized in Appendix S1.

For our systematic review, we opted to include multiple observations from individual papers where appropriate. For example, many studies included multiple microbial treatments (e.g., different microbial taxa/strains). Observations were considered independent only if they (1) considered a different microbe, insect, or plant host or (2) took place in unique environments (i.e., biologically unique locations or different years). When multiple concentrations of the same microbial inoculant were examined, we focused on the highest dose that was recorded. In addition, when the impact of microbes was examined with and without environmental stresses, we focused on the unstressed treatment group. When different intensities or durations of herbivore feeding were imposed, the most intense or longest treatment was selected. Finally, when multiple measurements were taken over time, we used the latest recorded measurement.

### Categorizing microbial contributions to plant expression of tolerance

We applied our conceptual framework for describing microbe‐mediated tolerance (Figure 1; outlined in Appendix S2) to categorize all observations from studies included in our systematic review. This framework compares plant fitness in the presence and absence of both microbes and herbivory (i.e., four treatment groups). It should be noted that tolerance is best measured by directly comparing whole‐plant fitness (e.g., yield). Tolerance, or the amelioration of fitness loss, may or may not be accurately reflected when using individual fitness components or proxies of tolerance (e.g., biomass, photosynthetic capability). However, few papers in our systematic review measured yield (*n* = 7 papers); therefore, we apply our framework to both plant fitness and proxies, such as root or shoot biomass.

In general, to assess microbial involvement in tolerance, a fully crossed experimental design consisting of two main treatments (±microbe and ±insect) is ideal, but partial designs (e.g., missing a “+microbe/−insect” group) can be evaluated with some generalizations using our framework. For example, when a partial design lacks an uninfested holobiont treatment, the magnitude of microbial contributions can still be inferred, though whether effects occur through constitutive or induced pathways cannot. Observations from partial design were considered “uncategorized tolerance” or “no effect.”

When studies include a fully crossed experimental design, fitness comparisons are made with the control group, which are uninfested host plants that have not received a microbial inoculant (“−microbe/−insect”). Microbial‐mediated tolerance responses are therefore largely possible when herbivory decreases host plant fitness, or in other words when there is a “cost of herbivory” in the absence of microbial inoculants (Figure 1A, red dashed line). In these cases where the host plant is susceptible to herbivory, fitness comparisons between the infested holobiont (+microbe/+insect), infested host plant (−microbe/+insect), and control (−microbe/−insect) can tbe used to characterize the magnitude of microbial contribution to host tolerance. For example, microbial treatments can facilitate *compensation* if infested holobiont fitness matches control fitness. In this case, the cost of herbivory is overcome by the addition of a microbial treatment. Microbes can also contribute to tolerance through *overcompensation* (+microbe/+insect > control) or *partial compensation* (control < +microbe/+insect > −microbe/+insect) (Figure 1A). Microbes can also have *no effect* on host tolerance (+microbe/+insect = −microbe/+insect) or further exacerbate susceptibility/non‐tolerance by decreasing fitness below the cost of herbivory alone (+microbe/+insect < −microbe/+insect < control).

Microbial‐mediated tolerance effects can be further categorized as occurring through constitutive or induced pathways by comparing both infested and uninfested holobiont treatments (Figure 1 “microbe + insect” vs. “microbe alone”), and depends on whether uninfested holobiont fitness is greater (constitutive), equal, or less than (induced) that of control plants (Figure 1B). Microbial impacts on tolerance can occur constitutively where the addition of the microbe(s) is always beneficial for plant fitness, or in other words can be seen regardless of whether or not the plant has experienced herbivory (Figure 1B, top panel). For example, when a microbial treatment helps the host plant compensate for fitness losses to herbivory (+microbe/+insect = −microbe/−insect), this response could be considered constitutive if the uninfested holobiont also experiences improved fitness relative to the control. Alternatively, microbes affect tolerance responses through induced pathways when the addition of a microbial inoculant can either be negative or have no effect on host fitness compared to the control, but when in the presence of herbivory there is a boost to fitness (Figure 1B, bottom panel). For example, a microbial treatment could facilitate induced compensation if an un‐infested holobiont (“microbe alone”) showed no evidence of improved fitness, but a positive effect of microbial treatment is seen in response to herbivory in the infested holobiont group.

Finally, it is possible that herbivory either has no effect or increases host plant fitness in the absence of microbial inoculants (i.e., insect alone ≥ control). In these cases of natural host plant tolerance where this is no observed cost to herbivory, microbial treatments could either have no effect (i.e., only host genetics contributes to tolerance), further increase (e.g., microbi‐mediated overcompensation) or decrease (e.g., microbial‐mediated susceptibility) infested holobiont fitness compared to infested host plant fitness.

### Meta‐analysis: Estimating microbial effects on plant tolerance

We first examined each paper included in our systematic review to assess how often measures of tolerance such as yield, biomass, oxidative stress, and photosynthetic components were evaluated to identify measures that could be used for a meta‐analysis. While all 40 papers considered tolerance as proxied by biomass, relatively few considered whole plant fitness (e.g., yield) or measures reflective of tolerance mechanisms such as oxidative stress and/or photosynthesis (Appendix S3: Figure S1). Because of the few data related to yield (*n* = 7 papers), oxidative stress, and photosynthesis, we focused the meta‐analysis on summarizing how the presence of one or more microbes influenced tolerance to herbivory as captured by plant shoot, root, reproductive, and/or total biomass.

We next extracted means and estimates of uncertainty (SD or SE) of biomass measures (shoot, root, reproductive, and/or total) from the infested host treatment group (i.e., “insect alone” or “+insect/−microbe”) and the infested holobiont treatment group (i.e., “microbe + insect” or “+insect/+microbe”) where a microbial inoculant was applied to the host plant and then exposed to herbivory. These parameters were taken from publicly available data or tables whenever possible, and estimated from figures with WebPlotDigitizer when necessary. We were unable to extract biomass measures from five of the 40 papers due to either (1) insufficient detail being provided about estimates of uncertainty, (2) figures not depicting the treatments of interest, or (3) biomass being presented as percent regrowth. These five excluded papers are noted in Appendix S1.

From each paper included in our meta‐analysis (*n* = 35), data on key insect, plant, microbial, and environmental attributes were collected for each independent observation. Insect‐focused traits included herbivore feeding guild and site of herbivore feeding. Microbial attributes included microbial functional guild, microbial inoculant source, microbial community species richness, and the effect of microbe treatment on insect fitness. Plant information included family, age at infestation, “regrowth period” (i.e., how long the plant was allowed to regrow after the herbivory treatment ended), and plant cultivation status (i.e., domesticated or wild). Finally, we collected information on the experimental environment, including whether experiments were conducted under controlled conditions or in the field. These data were later used as moderators in our meta‐analysis models to test how the strength of microbial‐mediated expression of tolerance responses changed according to key insect, microbe, and plant traits (e.g., different insect feeding guilds). Collectively, these attributes were chosen based on existing research demonstrating or predicting differences in the expression of tolerance or impacts on underlying tolerance mechanisms across identified levels/categories (e.g., chewers vs. phloem‐feeders, wild vs. crops, growth‐promoting root microbes).

We estimated effect sizes that capture microbial impacts on plant tolerance to insect herbivory with Hedges' *d* using the metafor package (v. 4.2‐0) (Viechtbauer, 2010) in R (v. 4.2.3) (R Core Team, 2021). The standardized mean difference was used (measure = “SMD”), meaning that Hedges' *d* was calculated as follows:

$$d=\frac{\mu_{1}-\mu_{2}}{\sqrt{\frac{\left(n_{1}-1\right)\times {\sigma_{1}}^{2}+\left(n_{2}-1\right)\times {\sigma_{2}}^{2}}{n_{1}+n_{2}-2}}}$$

where all parameters with the subscript 1 refer to the “microbe + insect” treatment (i.e., “+microbe/+insect”), while parameters with the subscript 2 refer to the “insect alone” treatment (i.e., “−microbe/+insect”). A positive effect size indicates that, relative to the “−microbe/+insect” treatment, the “+microbe/+insect” treatment improved plant biomass under herbivory. An effect size of zero reflects equal biomass between the “+microbe/+insect” and “−microbe/+insect” treatment (i.e., fitness loss to herbivory was the same in the presence and absence of microbial treatments). Finally, a negative effect size indicates a microbial treatment contributed to greater susceptibility or non‐tolerance under herbivory.

To avoid issues related to nonindependence stemming from multiple biomass measurements (e.g., root and shoot biomass) taken from the same plant, we calculated composite effect sizes with the aggregate.escalc function at the observation level. Rho, the correlation of sampling errors of measurements from the same plant, was estimated by averaging the correlation coefficients of pairwise comparisons of root, shoot, total, and reproductive biomass.

First, we estimated the overall effect of root microbial treatments on tolerance to insect herbivory across all observations and studies. To do so, we fit a random‐effects model to our composite effect sizes with the rma.mv function with the default restricted maximum likelihood estimator and the following random effects structure: Observation (i.e., an observation‐level effect) nested within Experiment (i.e., unique experiments within Citations, defined as unique by the presence of separate controls), nested within Citation (i.e., individual papers). In addition, we calculated *I* ^2^, an estimate of heterogeneity in effect size outcomes, to estimate what percent of heterogeneity could be associated with each of our random effects: Citation, Experiment, and Observation.

Next, we examined how microbial‐mediated tolerance responses varied with key attributes of insects, plants, and root microbial communities by fitting separate random‐effects models to each of our eight categorical moderators (herbivore feeding guild, site of herbivore feeding, plant cultivation status, microbial functional guild, microbial inoculant source, experimental environment, microbial community species richness, and effect of microbe treatment on insect fitness) and one continuous moderator (duration of regrowth). This meta‐regression approach was selected to enable the inclusion of observations with missing information for one or more moderators, which in our dataset equated to 47 of 134 total observations across the 35 papers that met inclusion criteria for meta‐analysis. Moderator information was collected for eight of the nine moderators based on the authors' descriptions of experimental methods. These moderators included the following information: (1) herbivore feeding guild (chewing, piercing‐sucking, galling, or mixed herbivore community, where the latter was used in cases of herbivory from insects of multiple feeding guilds); (2) site of herbivore feeding (shoot, root, or whole plant); (3) plant cultivation status (cultivated or uncultivated); (4) microbial functional guild (arbuscular mycorrhizal fungi [AMF], N‐fixing bacteria, other plant growth‐promoting fungi [PGPF], other plant growth‐promoting bacteria [PGPB], or mixed, with the latter used when microbes from multiple functional guilds were examined); (5) microbial inoculant source (lab stock [i.e., isolates from public or private labs or repositories], field isolate, or commercial product); (6) experimental environment (growth chamber, greenhouse, experimental station, agricultural field, or “other field” used to describe miscellaneous spaces such as student gardens or otherwise unidentified outdoor spaces); and (7) duration of regrowth (or number of days between the end of herbivory treatments and the collection of biomass measurements). The eighth moderator—total soil community diversity—was used to capture the number of microbial species or strains a treated plant's roots were exposed to from either inoculants or natural field‐collected communities. This moderator was characterized as either a single species (when one strain was inoculated into sterile soil), small consortia (when a mix of selected strains were introduced into sterile soil), and otherwise complex (when unsterilized soil or strains introduced into unsterilized soil were used). The ninth and final moderator was based on experimental results—the effect of microbes on herbivore fitness—and was either positive when one or more significant and positive effects of microbial treatments on insect fitness traits (e.g., larval growth rate) were reported; negative when one or more negative effects were reported; or neutral when consequences for insect fitness were considered but no significant effects were reported. In a few observations, both positive and negative effects on insect fitness were reported, but the number of these mixed observations was too low (<5) for the level to be included in moderator analyses.

Finally, to examine whether any one study exerted particularly strong influence over our results, we conducted a leave‐one‐out analysis. This analysis involved iteratively removing one Citation from our dataset and re‐running our main model. The grand mean effect sizes produced by this leave‐one‐out analysis were then visualized to help us identify particularly influential papers. This visualization was supplemented with estimates of Cook's distance (*D*) to help identify influential Citations, with cutoffs of *D* > 0.5 and *D* > 1 considered. In addition, to estimate the extent to which publication bias might be influencing our results, we created a funnel plot and conducted an Egger's‐type test by refitting our original model with variance as a moderator. Finally, to estimate how robust the results of this meta‐analysis were against the consequences of publication bias, we also calculated a fail‐safe N, or the number of unpublished papers with null results needed to render our grand mean effect size nonsignificant. All figures were made using the ggplot2 package (v. 3.4.2) (Wickham, 2016) in R and Inkscape. All data files and code for the literature review and meta‐analysis, including all statistical analyses and data visualization, are available in Dryad (Tronson & Enders,  2024a) and Zenodo Tronson & Enders, 2024b).

## RESULTS

### Systematic review of studies examining root microbial effects on plant tolerance to herbivory

**FIGURE 2:**
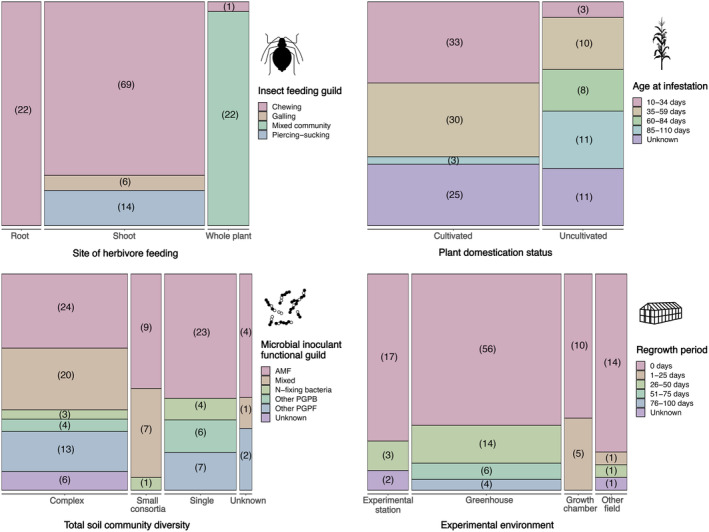
Mosaic plots summarizing observations from papers included in this systematic review across key insect, plant, microbe, and experimental attributes. In each panel, two traits are visualized, with bar width on the *x*‐axis and bar height on the *y*‐axis reflecting the number of observations from this meta‐analysis that examined these insect, plant, microbe, and experimental environment traits. The numbers in parentheses represent the number of observations for which the combination of two traits is considered. For example, the largest mosaic in the lower right‐hand panel shows that 56 observations both took place in a greenhouse and allowed plants no regrowth period after herbivory. Icons from the NounProject. AMF, arbuscular mycorrhizal fungi; PGPB, plant growth‐promoting bacteria; PGPF, plant growth‐promoting fungi.

Of the 293 papers returned in our literature search, 40 met the inclusion criteria. These papers comprised 134 observations (average of 3.4, range of 1–15 observations/paper), where separate observations within the same paper were distinguished by a unique microbe, insect, plant, or environmental component (Figure 2, Appendix S1, Appendix S3: Figure S1).

While most experiments evaluated the presence or absence of a single microbial strain (54% of observations), these single strains were often examined within more complex soil communities. When we considered total soil community diversity, we found that most observations (52%) considered relatively high‐diversity communities in which treatments involved the transfer of either a whole microbial community (8%) or, more often, single strains or consortia that were introduced into intact communities (i.e., unsterilized noncommercial soil) (Figure 2). These microbial treatments were derived from a balanced variety of sources, with 29% originating from academic stock (e.g., from laboratories or repositories), 37% being commercial amendments, and 20% being field isolates. In the case of single strains or small consortia, most treatments utilized fungi (61% of observations) and especially AMF (45% of observations). The remaining non‐AMF fungi were usually entomopathogenic fungi with endophytic, plant growth‐promoting properties (e.g., *Beauveria bassiana*) or other PGPF (e.g., *Piriformospora indica*). On the other hand, just 18 observations (13%) focused on bacteria, with 10 utilizing a broad group of PGPB and the remaining eight observations considering nitrogen‐fixing bacteria.

With regard to insect traits of interest, more than half of the 134 observations (51%) focused on aboveground chewing herbivores, while root‐feeding herbivores and piercing‐sucking insects were comparatively underrepresented (16% and 10% of observations, respectively) (Figure 2). In addition, the effect of microbial treatments on insect herbivore fitness varied. Tolerance defenses are generally assumed to avoid challenging insect fitness (Espinosa & Fornoni, 2006; Stowe et al., 2000; Strauss & Agrawal, 1999) and indeed, of the studies that considered how microbial treatments impacted insect fitness, most reported that herbivore fitness was unaffected (54%) or increased (20%). However, microbial additions occasionally decreased herbivore fitness (22%), suggesting that some microbial treatments may have influenced plant performance in part through improving resistance.

Opportunities for compensatory responses, an often discussed component of plant tolerance, albeit overwhelmingly in the context of herbivory from chewers (Koch et al., 2016), were somewhat well afforded in experimental designs, as 25% of observations designated an insect‐free regrowth or compensatory period after herbivore treatments. The average age at which plants were challenged with herbivory was 47 days after germination, though infestation tended to occur earlier in cultivated plants (37 days) than in uncultivated plants (69 days). Even though most (68%) observations considered cultivated plants, just 4% of observations took place in agricultural fields, with an additional 16% being on experimental stations. Instead, most experiments were conducted in climate‐controlled, indoor environments (greenhouses or growth chambers; 71%).

While all 40 papers reported biomass measures that could be adopted as proxies for plant fitness, only eight papers considered oxidative stress mitigation and/or photosynthetic parameters (Appendix S3: Figure S1), both of which are important domains of plant tolerance to insect herbivory (Koch et al., 2016). Because of the shortage of available research on how microbes mediate these two components of tolerance, we opted to focus the subsequent meta‐analysis on biomass‐based estimates of plant fitness.

### Meta‐analysis: Root microbial effects on plant biomass under herbivory

Five of the 40 papers that fit the inclusion criteria could not be included in the meta‐analysis, largely due to issues with how estimates of uncertainty were presented (e.g., missing error bars, error bars not specified as SD or SE). The remaining 35 papers were included in the meta‐analysis and contributed 113 observations, for an average of 3.2 observations per paper.

**FIGURE 3:**
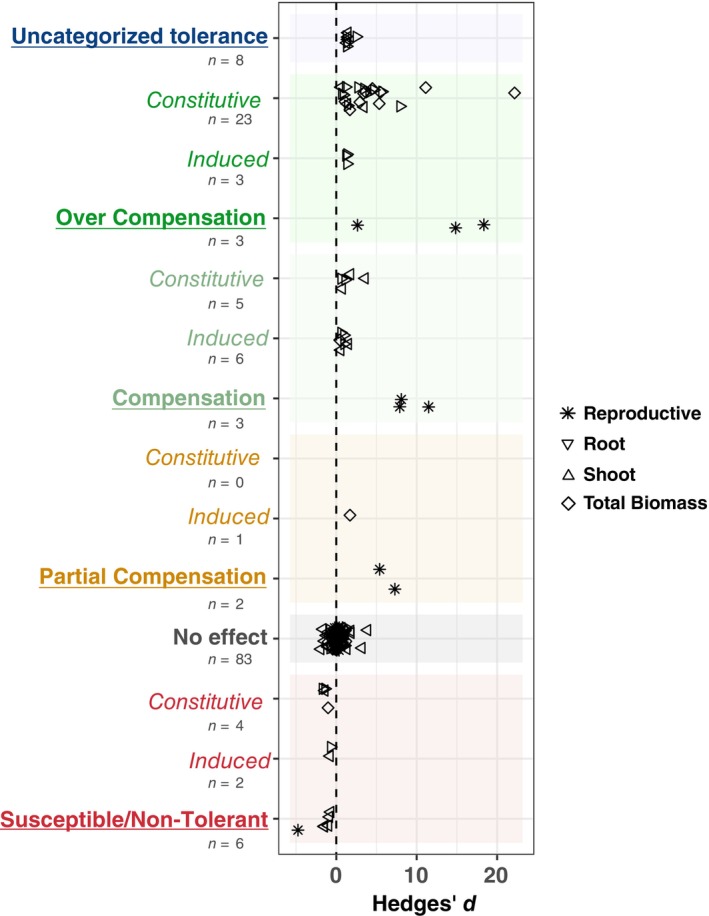
Summary of the range in microbial‐mediated tolerance responses. Tolerance categories (*x*‐axis) assigned using our proposed framework (Figure 1) are overlaid with effect sizes (*y*‐axis) estimating impact of microbes on expression of tolerance to herbivory using biomass measures (plant shoot, root, reproductive, total biomass) as a fitness proxy. Shaded boxes delineate the tolerance categories (i.e., uncategorized tolerance, overcompensation, compensation, partial compensation, no effect, and susceptibility/non‐tolerance). Observations within categories are separated by type (induced vs. constitutive) where possible. Effect sizes were estimated using Hedges' *d*—where a positive effect size indicates that the addition of a microbial inoculant improved plant fitness under herbivory (i.e., improved tolerance) for all papers included in our systematic review of the literature.

First, we applied our conceptual framework (Figure 1) to categorize all observations included in our meta‐analysis. Our tolerance categorization framework utilizes comparisons between the infested holobiont “microbe + insect” treatment and each of the three other treatments from a fully crossed experimental design. One strength of this approach is the ability to tease apart host plant genetic and microbial contributions to the expression of tolerance (see Appendix S2: Flowchart and key). Our goal was to determine how well our framework aligned with the effect sizes (i.e., Hedges' *d*), which were estimated by comparing biomass measures in both infested treatments (i.e., “microbe + insect” and “insect alone”). Overall, these two measures generally aligned, with all instances of susceptibility associating with negative effect sizes and all instances of partial compensation, compensation, and overcompensation associating with positive effect sizes (Figure 3). Observations associated with no effect of microbes (59%) had a relatively even spread of effect sizes around 0 (median = 0.15; range = −1.94–3.87). For cases where the addition of microbes reduced the cost of herbivory, there was a range of positive effects sizes [0.44; 18.36], with overcompensation (18%) having highest representation, followed by compensation (7%), uncategorized tolerance (4%), and partial compensation (3%) (Figure 3). Among these observations, we also found evidence of microbial amendments improving tolerance not only through general plant growth‐promoting mechanisms (i.e., constitutively) but also through induced pathways. In cases of induced tolerance, the positive effects of a microbial treatment on plant fitness were not seen in the “microbe alone” treatment but were revealed when the plant and its root microbes were exposed to herbivory (i.e., infested holobiont or “+microbe/+insect” treatment). Finally, we found evidence for natural host plant tolerance where there was no cost to herbivory or an increase in biomass (*n* = 57), entirely microbial‐derived tolerance where addition of microbes significantly reduced the cost to herbivory (*n* = 26/48 cases where host plant was susceptible), and combined host plant and microbial tolerance (*n* = 20).

**FIGURE 4:**
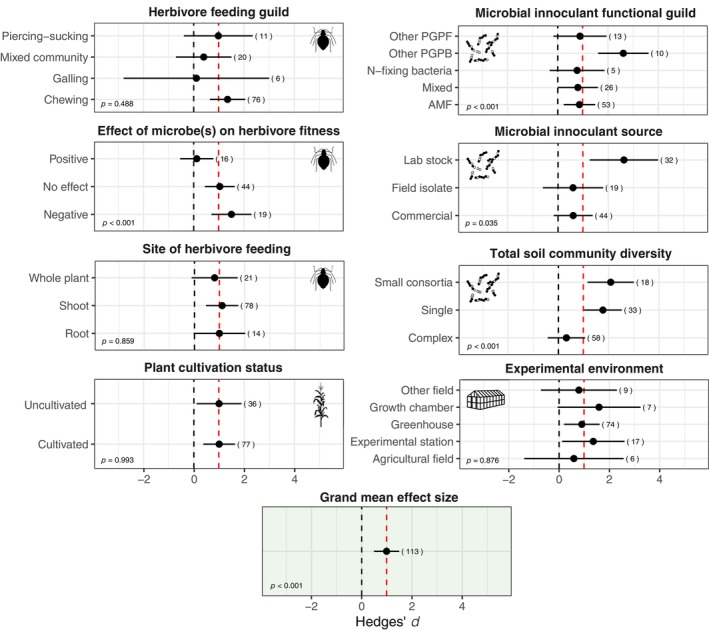
Moderator analysis of the impacts of key insect, microbial, plant, and experimental attributes on the expression of microbial‐mediated tolerance. Positive effect sizes (i.e., Hedges' *d*) indicate the infested holobiont treatment (“microbe + insect”) either overcame a fitness loss to herbivory (control = infested holobiont > infested uninoculated) or had greater fitness than the uninfested control (infested holobiont >control and >infested uninoculated), which demonstrates that the addition of a microbial treatment improved plant tolerance to herbivory. The red vertical dashed line represents the grand mean effect size estimated across all observations included in this meta‐analysis, while the black vertical dashed line represents an effect size of zero (i.e., no impact of a microbe on tolerance). Each panel contains the results of one of our moderator analyses, with each horizontal bar representing a level within that moderator. Numbers in parentheses refer to the number of observations representing a given moderator level. Significant *p* values indicate that a given moderator was able to explain variation in tolerance effect sizes. Icons from the NounProject. AMF, arbuscular mycorrhizal fungi; PGPB, plant growth‐promoting bacteria; PGPF, plant growth‐promoting fungi.

In our meta‐analysis, the grand mean effect size estimated across all observations was 0.99 [0.49; 1.49], indicating a strong reported effect of microbial treatments on improving host fitness under herbivory (*z* = 3.87, *p* < 0.001) via increased vegetative and reproductive biomass (Figure 4). This strong and positive grand mean effect size indicates that, on average, a beneficial microbial treatment increases plant biomass under herbivore stress, thus improving tolerance by ~1 SD. Estimates of *I* ^2^ revealed a substantial amount of unexplained heterogeneity: 94.51%. Of this heterogeneity, 25.08% existed between levels of Citation, while a dominant 59.91% came from the Experiment level, which controlled for multiple comparisons with the same control. The remaining 9.52% could be attributed to sampling variance. As expected, our funnel plots and Egger's‐type test indicated evidence of publication bias, as variance explained a significant amount of heterogeneity among the collected observations when included as a moderator in a random effects model (*Q*_*M*_ = 67.66, *p* < 0.001). Specifically, this indicates that observations with greater variance (e.g., potentially observations with few replicates) were associated with larger effect sizes. However, the fail‐safe N was calculated as 4730 observations, and when divided by the number of observations per paper (3.2), this calculation suggests that 1478 papers with null results would be needed to render the positive and significant grand mean effect size presented here nonsignificant. Overall, this points to the general robustness of these results, despite potential publication bias.

Our sensitivity or leave‐one‐out analysis suggested that one paper with a Cook's *D* of 1.17 that was contributing six observations was likely influencing our results. When this paper was removed from the dataset, the grand mean effect size was 0.71 [0.33; 1.09] (Appendix S3: Figure S2). This paper was the only Citation with a Cook's *D* > 0.5. To ensure that this paper was not determining certain moderator results, we reran meta‐regression analyses with the observations from this paper excluded from the dataset and report results from both models (full and leave‐one‐out adjusted).

Our meta‐regression analyses evaluated the extent to which eight categorical moderators (Figure 4) and one continuous moderator could explain heterogeneity in our random effects model. All three moderators characterizing microbial treatments were able to explain patterns in the collected effect sizes estimating microbial impacts on tolerance as proxied by vegetative and reproductive biomass (Figure 4). For one, total soil community diversity, which characterized the species richness of the microbial community with which plant roots interfaced, significantly explained variation in plant biomass under herbivory (*Q*_*M*_ = 17.06, *p* < 0.001). Complex communities, which were treatments of either whole soil microbial communities or single strains or consortia introduced into intact soil communities, were associated with poorer microbial‐mediated tolerance responses (*d* = 0.32 [−0.42; 1.06]). On the other hand, single strains (i.e., species richness of 1) and small consortia were associated with 79% and 110%, respectively, greater improvements in biomass under herbivory than in the grand mean effect size. Microbial inoculant functional guild also influenced microbial‐mediated tolerance, as determined by biomass (*Q*_*M*_ = 19.76, *p* < 0.001). This effect appeared to be driven by PGPB, which were associated with biomass improvements that were more than twice as large as those of the grand mean effect size (*d* = 2.61 [1.60; 3.61]). However, this group comprised just 10 observations from four papers. Finally, when assessing how the source of inoculants affected microbial‐mediated tolerance, we found that laboratory stock (i.e., isolates maintained within laboratories, repositories, or other institutions) supported marginally significant biomass improvements under herbivory compared to commercial formulae or field isolates (*Q*_*M*_ = 6.70, *p* = 0.04; lab stock: *d* = 2.62 [1.26; 3.98]). However, when we reran meta‐regression analyses without the influential paper identified in our leave‐one‐out analysis, we found that this effect of microbial source was no longer significant (*Q*_*M*_ = 1.22, *p* = 0.54).

Neither herbivore feeding guild (*Q*_*M*_ = 2.43, *p* = 0.49) nor site of herbivore feeding (*Q*_*M*_ = 0.30, *p* = 0.86) was able to explain patterns in effect sizes (Figure 4). However, the effect of microbes on herbivore fitness did affect the strength of microbial‐mediated tolerance responses (*Q*_*M*_ = 20.54, *p* < 0.001). When microbial treatments improved insect fitness, tolerance effect sizes tended to be lower (*d* = 0.12 [−0.54; 0.78]), whereas more positive effect sizes were seen among studies reporting that microbial treatments negatively affected herbivore fitness (*d* = 1.50 [0.70; 2.30]). When considering plant traits, we found little evidence that the ability of microbial treatments to support tolerance to herbivory differed between cultivated and uncultivated plants (*Q*_*M*_ < 0.001, *p* = 0.99). In addition, both the continuous moderator duration of insect‐free regrowth allowed to plants after infestation (*Q*_*M*_ = 0.08, *p* = 0.77) and the categorical moderator experimental environment (*Q*_*M*_ = 1.21, *p* = 0.88) failed to impact microbe‐mediated tolerance.

## DISCUSSION

By developing and applying a novel conceptual framework for classifying how microbes mediate plant tolerance responses (Figure 1), we were able to capture the diversity of consequences microorganisms can have for plant defense—ranging from susceptibility to overcompensation (Figure 3). Our meta‐analysis revealed an overall strong and positive impact of root microorganisms on tolerance to herbivory as approximated by plant biomass measures; this effect was influenced most by microbial traits, including functional guild, inoculant source, and total soil community diversity, as well as the extent to which microbial treatments impacted herbivore fitness (Figure 4). Taken together, these results have implications for understanding the challenges of scaling microbial inoculants to field applications and the interconnectedness of resistance and tolerance within plant defenses. Finally, this synthesis highlights the paucity of research on plant–microbe interactions as they relate to tolerance to herbivory, with non‐AMF microorganisms, phloem‐feeding insects, and components of tolerance related to photosynthesis, oxidative stress, and hormone signaling receiving particularly poor coverage.

### Microbes can improve tolerance through both induced and constitutive avenues

This meta‐analysis is the first to establish a role for root microbes in the expression of plant tolerance to herbivore damage, specifically in the form of compensatory growth (e.g., compensation, overcompensation) that counteracts the cost of herbivory. Evidence for compensatory growth in response to herbivory is well established (Garcia & Eubanks, 2019); however, our results suggest these previously documented effects are likely to be attributed in part to microbes (e.g., AMF or other beneficial symbionts). Previous studies documenting compensatory growth typically define the control as “uninfested” or “un‐damaged” plants, which are then compared to the experimental group of insect infested/damaged plants. In most documented cases of overcompensation/compensation, the holobiont (host plant + soil microbes) is used in experiments. In contrast, our meta‐analysis examined tolerance from experiments that included a “no microbe” control treatment (e.g., sterile soil). Although we find more cases of constitutively expressed microbial‐mediated overcompensation than other types of tolerance (Figure 4), additional experiments incorporating “no microbe” controls are needed to further tease apart the relative contributions of host and microbes to holobiont tolerance expression in systems where these defense responses have been previously characterized. We found microbes tended to boost tolerance more often when there was cost to herbivory/host plant was susceptible (26/48–54% of cases) compared to cases where natural host plant tolerance was present (20/57–35% of cases). It will be particularly important to examine cases where the host plant is naturally tolerant (i.e., low to no fitness cost to herbivory when microbes absent) to determine whether microbes generally enhance tolerance or primarily in cases where the host is already susceptible to herbivory.

Though microbially induced tolerance has been noted here and by others (e.g., Cosme et al., 2016), the mechanisms through which microbes induce tolerance are poorly understood. We identified several cases of microorganisms inducing tolerance to herbivory, which occurs when the effects of a microbial treatment on plant biomass are absent in uninfested treatments and instead appear only in response to herbivory (Bernaola & Stout, 2021; Currie et al., 2011; Eichholtzer et al., 2021). Among papers reviewed here, most cases of induced tolerance involved especially strong increases in antioxidant enzyme activities (Chen et al., 2022; Metwally et al., 2022; Selvaraj et al., 2020) or photosynthetic efficiency (Chen et al., 2022; Wang et al., 2022) in response to herbivory. However, one of the clearest cases of induced tolerance relates to biomass and comes from Cosme et al. (2016), who reported that a fungal endophyte was able to induce tolerance to above‐ and belowground herbivory by suppressing herbivore‐induced surges in jasmonic acid, which can limit root growth. Instead, endophyte treatments were associated with improved gibberellin (GA) synthesis that ultimately favored plant investment in root growth.

Studies also noted constitutive expression of microbial‐mediated tolerance, which occurs when a microbial treatment improves plant growth in both the absence and presence of herbivory. Hormones such as auxins, especially indole‐3‐acetic acid, or ACC deaminase are known to be involved in the microbial‐mediated promotion of plant growth in the absence of herbivore infestation (Backer et al., 2018; Sukumar et al., 2013). However, more research is needed to determine whether the same pathways can promote root growth in plants experiencing herbivory, as plant growth promotion does not always translate into improved tolerance. Some microbes have been shown to improve plant growth in the absence of herbivores, but offer no such benefit under insect herbivory (Gange et al., 2002; Zitlalpopoca‐Hernandez et al., 2017). In addition, plants amended with plant growth‐promoting microbes that facilitate nutrient uptake can become more desirable food sources and consequently experience more severe herbivory from phloem‐feeders in particular (Gange & West, 1994). This discrepancy underscores the importance of studying these plant growth‐promoting microbes explicitly under the context of tolerance responses to plant herbivory.

Although we find strong evidence for microbial‐mediated tolerance, there is also potential for root microbiomes to affect resistance and tolerance defenses simultaneously. Here, we found stronger effect sizes were associated with microbial inoculants that also negatively impacted herbivore fitness (Figure 4—“effect of microbes on herbivore fitness”). This relationship between negative effects on insect fitness and positive effects on our biomass‐based effect sizes could be caused by more resistant plants losing less biomass, which would obscure our ability to detect tolerance, or the simultaneous expression of plant resistance and tolerance. In cases where the addition of a microbial inoculant benefited the herbivore, microbial impacts on plant tolerance were near zero. While tradeoffs between resistance and tolerance have been detected in certain environments or plant–insect systems, evidence for this tradeoff being widespread has proven difficult to find (Leimu & Koricheva, 2006). Some authors have noted that microbes increased host plant tolerance by suppressing costly resistance mechanisms (e.g., Barazani et al., 2005), but others have found that microbial improvements to tolerance had no effect on the strength of resistance responses (Cosme et al., 2016). Further research is needed to untangle the complex interacting layers of microbial‐mediated defense.

### Microbial‐mediated improvements to tolerance are stronger in the absence of interspecific competition

Surprisingly, tolerance effect sizes did not differ according to the experimental environment (i.e., growth chamber, greenhouse, experimental station, agricultural field, or other field). This result fails to corroborate the many independent studies (Bacilio et al., 2017) and reviews (Zhang et al., 2019) that have reported weaker effects of microbial additions in field environments than in controlled indoor environments. However, this result may be clarified by the significant effect of soil community diversity on tolerance effect sizes (Figure 4). Though field experiments always involved plant exposure to relatively high‐diversity communities, greenhouse or growth chamber experiments sometimes also utilized unsterilized field soil as substrate. In these cases, the total soil community diversity was characterized as “Complex,” since any added inoculants had to compete with a relatively intact/natural microbial community to establish in the rhizosphere. These complex communities were associated with significantly lower tolerance effect sizes, with CIs for this group bracketing zero. In contrast, communities made up of either one (“Single”) or two or more selected strains (“Small consortia”) (i.e., inoculants in sterile soil) were associated with much stronger microbial‐mediated improvements to tolerance. Inoculants, which comprised most of this review's microbial treatments, may struggle to colonize a rhizosphere due to issues like poor motility that reduce a microbe's ability to compete with other soil microbes and establish in the rhizosphere, referred to as rhizosphere competence (Barret et al., 2011). Microbial inoculants may be most useful in controlled environments, while more nuanced and regionally specific approaches may be necessary to optimize defense‐enhancing microbial communities in open fields (French et al., 2021). To move toward field applications, we need more research understanding how soil type, climate, and other abiotic factors shape microbial communities, as well as research at the community level that elucidates how whole microbiomes contribute to plant defense and/or how single beneficial strains function in a diverse microbiome (Busby et al., 2017).

### Domestication of plants and microbes could impact tolerance to herbivory

In plants, domestication has been associated with altered root architecture and exudation patterns (Iannucci et al., 2017), reduced root microbiome diversity (Zachow et al., 2014), and an impaired ability to benefit from growth‐promoting microbes (Jaiswal et al., 2020). If certain crop ancestors evolved to recruit microbial communities that improve tolerance, this ability can become disrupted along the domestication process—intense selection for agriculturally relevant traits that is likely to have disregarded plant–microbe relationships (Porter & Sachs, 2020). However, in this study, cultivated and uncultivated plants did not differ in their ability to benefit from root microbial mutualists under herbivory (Figure 4). It is unclear whether this result applies broadly across crop domestication events, as observations from cultivated crops were dominated by the Poaceae family (*n* = 47/77) and primarily two species (rice, *n* = 12, and maize, *n* = 25), while Fabaceae (*n* = 14), Solanaceae (*n* = 10), Rosaceae (*n* = 3), and Cucurbitaceae (*n* = 3) were relatively less well represented. Furthermore, we had limited ability to investigate the broader role of plant functional group (e.g., forbs, grasses, degree of reliance on microbial mutualists, photosynthetic pathways) because of this disproportionate representation of a handful of plant families and several cases where all observations within a family came from a single study (e.g., Apocynaceae, Euphorbiaceae). Additional studies are needed that not only incorporate a wider range of crop‐wild ancestor pairings but also consider a greater diversity of ecological strategies, functional guilds, and growth forms.

Microbes can also be domesticated, for example, by maintaining cultures under long‐term artificial laboratory conditions or in cases of industrial application (Steensels et al., 2019). Our results showing similar effects of microbes on tolerance in wild and cultivated varieties could be due in part to the dominance of strains curated from commercial and laboratory sources, many of which have been put under artificial selection for desirable traits themselves and/or never coevolved alongside crop wild ancestors (Barreto et al., 2020; Steensels et al., 2019). While, in this meta‐analysis, microbes originating from field isolates performed no better than strains from commercial or laboratory sources, only some of these field isolates were selected for their coexistence with a wild crop ancestor (e.g., Zitlalpopoca‐Hernandez et al., 2017). Therefore, considering how plant–microbe coevolutionary relationships predict inoculant success would be a worthy area of future research.

### Conclusions and future directions

Overall, this review and meta‐analysis found strong evidence for root microbes enhancing plant tolerance to insect damage across a range of systems, particularly via compensatory growth mechanisms. However, existing research is disproportionately focused on (1) AMF, (2) chewing herbivores, (3) single strain inoculants rather than diverse communities, and (4) measurements of biomass when estimating microbial involvement in plant expression of tolerance to herbivory. More research into particularly understudied groups, such as phloem‐feeders or plant growth‐promoting rhizobacteria, is necessary in order to clarify our understanding of prevailing trends in microbial‐mediated tolerance and support the application of these relationships to improve crop health. Studies also tended to use vegetative biomass rather than yield or more comprehensive fitness measurements that properly assess tolerance levels. Future research combining both direct measures of fitness (e.g., yield) with individual traits linked to underlying tolerance mechanisms (e.g., biomass accumulation, photosynthetic activity) could more accurately indicate the potential for root microbes to modify plant tolerance to herbivory. More generally, increased research attention on microbial‐mediated tolerance defenses would enable follow‐up analyses that consider other key attributes, such as plant–insect coevolutionary relationships or plant functional groups, and take statistical approaches that allow for an understanding of interactions between moderators in meta‐analyses. In addition, considering variables such as the intensity of herbivore damage would allow us to understand how certain plant–microbe pairings manifest across the beneficial‐commensal‐parasitic continuum. Finally, the design of this meta‐analysis was such that observations were restricted to soil amendments, where a no‐microbe control could be established. Therefore, we did not consider top‐down approaches to creating beneficial soil microbial communities, such as crop and pest management approaches that impact soil and rhizosphere communities (e.g., cover cropping or intercropping), which deserve further analysis. As demand for more resilient and sustainable agricultural practices rises, an improved understanding of microbial‐mediated plant tolerance would open avenues to potentially long‐lasting, evolutionarily sustainable improvements to agricultural and rangeland practices (Peterson et al., 2018; Vannette & Hunter, 2009).

## AUTHOR CONTRIBUTIONS

Laramy Enders and Emily Tronson designed the study. Emily Tronson performed the literature review and collected data for the meta‐analysis. Laramy Enders and Emily Tronson performed all statistical analyses and data visualization. Emily Tronson and Laramy Enders wrote the complete first draft of the manuscript and both authors contributed to several rounds of revisions.

## CONFLICT OF INTEREST STATEMENT

The authors declare no conflicts of interest.

## Supporting information


Appendix S1.

Appendix S2.

Appendix S3.

## Data Availability

Data (Tronson & Enders, 2024a) are available in Dryad at https://doi.org/10.5061/dryad.v15dv425n. Code (Tronson & Enders,  2024b) is available in Zenodo at https://doi.org/10.5281/zenodo.13769410.
