# Supplementary material for: Root microbes can improve plant tolerance to insect damage: A systematic review and meta‐analysis
Source: Ecology. 2025 Jan 21;106(1):e4502. doi: 10.1002/ecy.4502 (PMC11750633; doi:10.1002/ecy.4502)
Supplement: Supplementary file 1 — Appendix S1. [file ECY-106-e4502-s002.pdf]

Root microbes can improve plant tolerance to insect damage: A systematic review and meta-analysis. Emily Tronson, Laramy Enders. *Ecology*.

Appendix S1: List of papers included in the systematic review.

References in red text were excluded from the meta-analysis due to insufficient detail being provided regarding estimates of uncertainty, figures not depicting the treatments of interest, or biomass being presented as percent regrowth.

(Balog et al., 2017; Batool et al., 2022; Bennett & Bever, 2007; Bernaola & Stout, 2019, 2021; Borowicz, 1997, 2009; Brunner et al., 2015; Caccavo et al., 2022; Charters et al., 2022; Chen et al., 2022; Contreras-Cornejo et al., 2021; Cosme et al., 2016; Coy et al., 2019, 2020; Currie et al., 2011; Dabré et al., 2021; de Bobadilla et al., 2017; Du et al., 2022; Eichholtzer et al., 2021; Forlano et al., 2022; Frew et al., 2020; Gan et al., 2017; Gange et al., 2002; He et al., 2017; Kabaluk & Ericsson, 2007; Kula et al., 2004; Metwally et al., 2022; Orians et al., 2018; Prischmann-Voldseth et al., 2020; Raglin et al., 2022; Real-Santillán et al., 2019; Rivera-Vega et al., 2022; Selvaraj et al., 2020; Tao et al., 2016; Wang et al., 2022; Whyle et al., 2022; Yang et al., 2021; Zeng et al., 2022; Zitlalpopoca-Hernandez et al., 2017)

Balog, A., Loxdale, H. D., Bálint, J., Benedek, K., Szabó, K.-A., Jánosi-Rancz, K.-T., & Domokos, E. (2017). The arbuscular mycorrhizal fungus Rhizophagus irregularis affects arthropod colonization on sweet pepper in both the field and greenhouse. *Journal of Pest Science*, *90*(3), 935–946. https://doi.org/10.1007/s10340-017-0844-1

Batool, R., Umer, M. J., Wang, Y., He, K., Shabbir, M. Z., Zhang, T., Bai, S., Chen, J., & Wang, Z. (2022). Myco-Synergism Boosts Herbivory-Induced Maize Defense by Triggering Antioxidants and Phytohormone Signaling. *Frontiers in Plant Science*, *13*, 790504. https://doi.org/10.3389/fpls.2022.790504

Bennett, A. E., & Bever, J. D. (2007). MYCORRHIZAL SPECIES DIFFERENTIALLY ALTER PLANT GROWTH AND RESPONSE TO HERBIVORY. *Ecology*, *88*(1), 210–218. https://doi.org/10.1890/0012-9658(2007)88[210:MSDAPG]2.0.CO;2

Bernaola, L., & Stout, M. J. (2019). Effects of arbuscular mycorrhizal fungi on rice-herbivore interactions are soil-dependent. *Scientific Reports*, *9*(1), 14037. https://doi.org/10.1038/s41598-019-50354-2

Bernaola, L., & Stout, M. J. (2021). The effect of mycorrhizal seed treatments on rice growth, yield, and tolerance to insect herbivores. *Journal of Pest Science*, *94*(2), 375–392. https://doi.org/10.1007/s10340-020-01279-7

Borowicz, V. A. (1997). A fungal root symbiont modifies plant resistance to an insect herbivore. *Oecologia*, *112*(4), 534–542. https://doi.org/10.1007/s004420050342

Borowicz, V. A. (2009). Organic Farm Soil Improves Strawberry Growth But Does Not Diminish Spittlebug Damage. *Journal of Sustainable Agriculture*, *33*(2), 177–188. https://doi.org/10.1080/10440040802660087

Brunner, S. M., Goos, R. J., Swenson, S. J., Foster, S. P., Schatz, B. G., Lawley, Y. E., & Prischmann-Voldseth, D. A. (2015). Impact of nitrogen fixing and plant growth-promoting bacteria on a phloem-feeding soybean herbivore. *Applied Soil Ecology*, *86*, 71–81. https://doi.org/10.1016/j.apsoil.2014.10.007

Caccavo, V., Forlano, P., Mang, S., Fanti, P., Nuzzaci, M., Battaglia, D., & Trotta, V. (2022). Effects of Trichoderma harzianum Strain T22 on the Arthropod Community Associated with Tomato Plants and on the Crop Performance in an Experimental Field. *Insects*, *13*(5), 418. https://doi.org/10.3390/insects13050418

Charters, M. D., Durant, E. K., Sait, S. M., & Field, K. J. (2022). Impacts of aphid herbivory on mycorrhizal growth responses across three cultivars of wheat. *PLANTS, PEOPLE, PLANET*, *4*(6), 655–666. https://doi.org/10.1002/ppp3.10302

Chen, C.-Y., Huang, P.-H., Yeh, K.-W., & Wang, S.-J. (2022). Colonization of *Piriformospora indica* enhances insect herbivore resistance of rice plants through jasmonic acid- and

antioxidant-mediated defense mechanisms. *Journal of Plant Interactions*, *17*(1), 9–18. https://doi.org/10.1080/17429145.2021.2008031

Contreras-Cornejo, H. A., Macías-Rodríguez, L., Real-Santillán, R. O., López-Carmona, D., García-Gómez, G., Galicia-Gallardo, A. P., Alfaro-Cuevas, R., González-Esquivel, C. E., Najera-Rincón, M. B., Adame-Garnica, S. G., Rebollar-Alviter, A., Álvarez-Navarrete, M., & Larsen, J. (2021). In a belowground multitrophic interaction, Trichoderma harzianum induces maize root herbivore tolerance against Phyllophaga vetula. *Pest Management Science*, *77*(9), 3952–3963. https://doi.org/10.1002/ps.6415

Cosme, M., Lu, J., Erb, M., Stout, M. J., Franken, P., & Wurst, S. (2016). A fungal endophyte helps plants to tolerate root herbivory through changes in gibberellin and jasmonate signaling. *New Phytologist*, *211*(3), 1065–1076. https://doi.org/10.1111/nph.13957

Coy, R. M., Held, D. W., & Kloepper, J. W. (2019). Rhizobacterial treatments of tall fescue and bermudagrass increases tolerance to damage from white grubs. *Pest Management Science*, *75*(12), 3210–3217. https://doi.org/10.1002/ps.5439

Coy, R. M., Held, D. W., & Kloepper, J. W. (2020). Rhizobacterial treatment of bermudagrass increases tolerance to damage from tawny mole crickets (*Neoscapteriscus vicinus* Scudder). *Pest Management Science*, *76*(3), 1078–1084. https://doi.org/10.1002/ps.5618

Currie, A. F., Murray, P. J., & Gange, A. C. (2011). Is a specialist root-feeding insect affected by arbuscular mycorrhizal fungi? *Applied Soil Ecology*, *47*(2), 77–83. https://doi.org/10.1016/j.apsoil.2010.12.002

Dabré, É. E., Lee, S.-J., Hijri, M., & Favret, C. (2021). The effects of mycorrhizal colonization on phytophagous insects and their natural enemies in soybean fields. *PLOS ONE*, *16*(9), e0257712. https://doi.org/10.1371/journal.pone.0257712

de Bobadilla, M. F., Friman, J., Pangesti, N., Dicke, M., van Loon, J. J. A., & Pineda, A. (2017). Does drought stress modify the effects of plant-growth promoting rhizobacteria on an aboveground chewing herbivore?: Microbe-plant-insect interactions under drought. *Insect Science*, *24*(6), 1034–1044. https://doi.org/10.1111/1744-7917.12477

Du, E., Chen, Y., Li, Y., Zhang, F., Sun, Z., Hao, R., & Gui, F. (2022). Effect of arbuscular mycorrhizal fungi on the responses of Ageratina adenophora to Aphis gossypii herbivory. *Frontiers in Plant Science*, *13*, 1015947. https://doi.org/10.3389/fpls.2022.1015947

Eichholtzer, J., Ballina-Gómez, H. S., Gómez-Tec, K., & Medina-Dzul, K. (2021). Arbuscular mycorrhizal fungi influence whitefly abundance by modifying habanero pepper tolerance to herbivory. *Arthropod-Plant Interactions*, *15*(6), 861–874. https://doi.org/10.1007/s11829-021-09868-8

Forlano, P., Mang, S. M., Caccavo, V., Fanti, P., Camele, I., Battaglia, D., & Trotta, V. (2022). Effects of Below-Ground Microbial Biostimulant Trichoderma harzianum on Diseases, Insect Community, and Plant Performance in Cucurbita pepo L. under Open Field Conditions. *Microorganisms*, *10*(11), 2242. https://doi.org/10.3390/microorganisms10112242

Frew, A., Powell, J. R., & Johnson, S. N. (2020). Aboveground resource allocation in response to root herbivory as affected by the arbuscular mycorrhizal symbiosis. *Plant and Soil*, *447*(1–2), 463–473. https://doi.org/10.1007/s11104-019-04399-x

Gan, H., Churchill, A. C. L., & Wickings, K. (2017). Invisible but consequential: Root endophytic fungi have variable effects on belowground plant-insect interactions. *Ecosphere*, *8*(3), e01710. https://doi.org/10.1002/ecs2.1710

Gange, A. C., Bower, E., & Brown, V. K. (2002). Differential effects of insect herbivory on arbuscular mycorrhizal colonization. *Oecologia*, *131*(1), 103–112. https://doi.org/10.1007/s00442-001-0863-7

He, L., Li, C., & Liu, R. (2017). Indirect interactions between arbuscular mycorrhizal fungi and Spodoptera exigua alter photosynthesis and plant endogenous hormones. *Mycorrhiza*, *27*(6), 525–535. https://doi.org/10.1007/s00572-017-0771-2

Kabaluk, J. T., & Ericsson, J. D. (2007). *Metarhizium anisopliae* Seed Treatment Increases Yield of Field Corn When Applied for Wireworm Control. *Agronomy Journal*, *99*(5), 1377–1381. https://doi.org/10.2134/agronj2007.0017N

Kula, A. A. R., Hartnett, D. C., & Wilson, G. W. T. (2004). Effects of mycorrhizal symbiosis on tallgrass prairie plant-herbivore interactions: Mycorrhizal symbiosis and herbivory. *Ecology Letters*, *8*(1), 61–69. https://doi.org/10.1111/j.1461-0248.2004.00690.x

Metwally, R. A., Azab, H. Sh., Al-Shannaf, H. M., & Rabie, G. H. (2022). Prospective of mycorrhiza and Beauvaria bassiana silica nanoparticles on Gossypium hirsutum L. plants as biocontrol agent against cotton leafworm, Spodoptera littoralis. *BMC Plant Biology*, *22*(1), 409. https://doi.org/10.1186/s12870-022-03763-x

Orians, C. M., Gomez, S., & Korpita, T. (2018). Does mycorrhizal status alter herbivore-induced changes in whole-plant resource partitioning? *AoB PLANTS*, *10*(1). https://doi.org/10.1093/aobpla/plx071

Prischmann-Voldseth, D. A., Özsisli, T., Aldrich-Wolfe, L., Anderson, K., & Harris, M. O. (2020). Microbial Inoculants Differentially Influence Plant Growth and Biomass Allocation in Wheat Attacked by Gall-Inducing Hessian Fly (Diptera: Cecidomyiidae). *Environmental Entomology*, *49*(5), 1214–1225. https://doi.org/10.1093/ee/nvaa102

Raglin, S. S., Kent, A. D., & Ngumbi, E. N. (2022). Herbivory Protection via Volatile Organic Compounds Is Influenced by Maize Genotype, Not Bacillus altitudinis-Enriched Bacterial Communities. *Frontiers in Microbiology*, *13*, 826635. https://doi.org/10.3389/fmicb.2022.826635

Real-Santillán, R. O., del-Val, E., Cruz-Ortega, R., Contreras-Cornejo, H. Á., González-Esquivel, C. E., & Larsen, J. (2019). Increased maize growth and P uptake promoted by arbuscular mycorrhizal fungi coincide with higher foliar herbivory and larval biomass of the Fall Armyworm Spodoptera frugiperda. *Mycorrhiza*, *29*(6), 615–622. https://doi.org/10.1007/s00572-019-00920-3

Rivera-Vega, L. J., Grunseich, J. M., Aguirre, N. M., Valencia, C. U., Sword, G. A., & Helms, A. M. (2022). A Beneficial Plant-Associated Fungus Shifts the Balance toward Plant Growth over Resistance, Increasing Cucumber Tolerance to Root Herbivory. *Plants*, *11*(3), 282. https://doi.org/10.3390/plants11030282

Selvaraj, A., Thangavel, K., & Uthandi, S. (2020). Arbuscular mycorrhizal fungi (Glomus intraradices) and diazotrophic bacterium (Rhizobium BMBS) primed defense in blackgram against herbivorous insect (Spodoptera litura) infestation. *Microbiological Research*, *231*, 126355. https://doi.org/10.1016/j.micres.2019.126355

Tao, L., Ahmad, A., Roode, J. C., & Hunter, M. D. (2016). Arbuscular mycorrhizal fungi affect plant tolerance and chemical defences to herbivory through different mechanisms. *Journal of Ecology*, *104*(2), 561–571. https://doi.org/10.1111/1365-2745.12535

Wang, Y., Li, Y., Tian, Z., & Duan, T. (2022). Arbuscular Mycorrhizal Fungus Alters Alfalfa (Medicago sativa) Defense Enzyme Activities and Volatile Organic Compound Contents in Response to Pea Aphid (Acyrthosiphon pisum) Infestation. *Journal of Fungi*, *8*(12), 1308. https://doi.org/10.3390/jof8121308

Whyle, R. L., Trowbridge, A. M., & Jamieson, M. A. (2022). Genotype, mycorrhizae, and herbivory interact to shape strawberry plant functional traits. *Frontiers in Plant Science*, *13*, 964941. https://doi.org/10.3389/fpls.2022.964941

Yang, Q., Siemann, E., Harvey, J. A., Ding, J., & Biere, A. (2021). Effects of soil biota on growth, resistance and tolerance to herbivory in Triadica sebifera plants. *Geoderma*, *402*, 115191. https://doi.org/10.1016/j.geoderma.2021.115191

Zeng, M., Hause, B., van Dam, N. M., Uthe, H., Hoffmann, P., Krajinski, F., & Martínez-Medina, A. (2022). The mycorrhizal symbiosis alters the plant defence strategy in a model legume plant. *Plant, Cell & Environment*, *45*(12), 3412–3428. https://doi.org/10.1111/pce.14421

Zitlalpopoca-Hernandez, G., Najera-Rincon, M. B., del-Val, E., Alarcon, A., Jackson, T., & Larsen, J. (2017). Multitrophic interactions between maize mycorrhizas, the root feeding insect Phyllophaga vetula and the entomopathogenic fungus Beauveria bassiana. *Applied Soil Ecology*, *115*, 38–43. https://doi.org/10.1016/j.apsoil.2017.03.014
