# Supplementary material for: Root microbes can improve plant tolerance to insect damage: A systematic review and meta‐analysis
Source: Ecology. 2025 Jan 21;106(1):e4502. doi: 10.1002/ecy.4502 (PMC11750633; doi:10.1002/ecy.4502)
Supplement: Supplementary file 2 — Appendix S2. [file ECY-106-e4502-s003.pdf]

Root microbes can improve plant tolerance to insect damage: A systematic review and meta-analysis. Emily Tronson, Laramy Enders. *Ecology*.

Appendix S2: Flow chart and dichotomous key for tolerance categories.

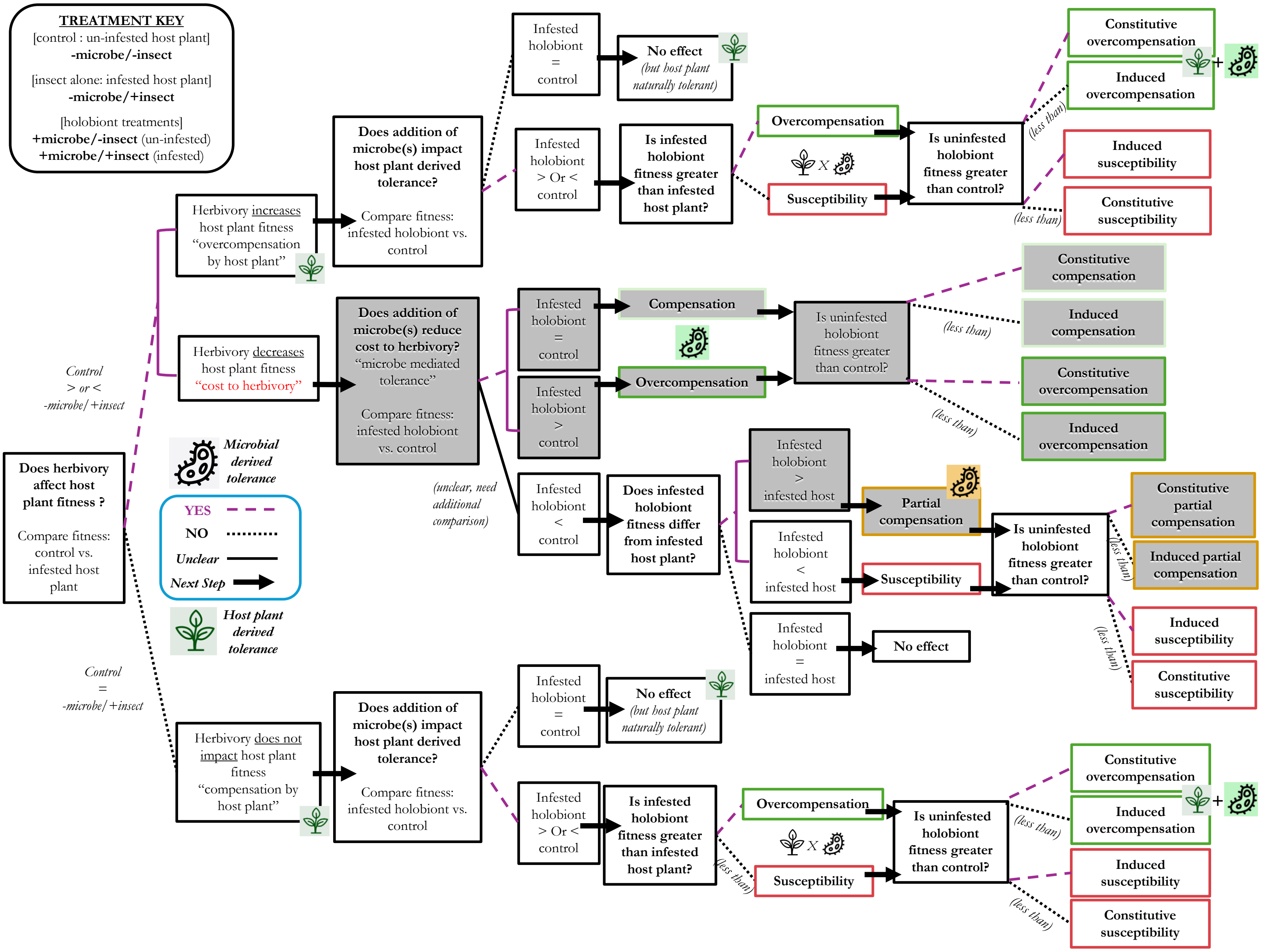

Flow Chart showing how tolerance categories are assigned based on comparisons across treatment groups – using the dichotomous key detailed below**.** This tolerance category framework differentiates between cases where the host plant has some level of natural tolerance to herbivory (e.g., no effect of herbivory or positive effect) compared to cases where microbes are involved in the expression of tolerance – shown using plant and microbe icons. It is possible for tolerance to be entirely host plant derived (plant icon), entirely microbial-derived (microbe icon) or a combination of both (plant + microbe icon). Grey shading highlights paths where tolerance is entirely microbially derived (i.e., host is susceptible, cost to herbivory).

Dichotomous Key for microbial contributions to plant tolerance

1. Herbivory affects host plant fitness (i.e., -microbe/-insect fitness > or < -microbe/+insect fitness) 2
1. Herbivory does not affect host plant fitness (i.e., -microbe/-insect fitness = -microbe/+insect fitness)- host plant tolerance/compensation 7

2. Herbivory decreases host plant fitness - cost to herbivory 3
2. Herbivory increases host plant fitness - host plant tolerance/overcompensation 9

3. Infested holobiont plant fitness equals uninfested host plant fitness .... *Compensation;* go to 1a
3. Infested holobiont plant fitness differs from uninfested host plant fitness 4

4. Infested holobiont plant fitness is greater than uninfested host plant fitness .... *Overcompensation;* go to 1a
4. Infested holobiont plant fitness is less than uninfested host plant fitness 5

5. Infested holobiont plant fitness equals infested host plant fitness .... *No effect of microbes on plant tolerance*
5. Infested holobiont plant fitness differs from infested host plant fitness 6

6. Infested holobiont plant fitness is greater than infested host plant fitness .... *Partial compensation;* go to 1a
6. Infested holobiont plant fitness is less than infested host plant fitness .... *Susceptibility;* go to 1b

7. Infested holobiont plant fitness equals uninfested host plant fitness .... *No effect of microbes on plant tolerance*
7. Infested holobiont plant fitness differs from uninfested host plant fitness 8

8. Infested holobiont plant fitness is greater than infested host plant fitness .... *Overcompensation;* go to 1a
8. Infested holobiont plant fitness is less than infested host plant fitness .... *Susceptibility;* go to 1b

9. Infested holobiont plant fitness equals uninfested host plant fitness .... *No effect of microbes on plant tolerance*
9. Infested holobiont plant fitness differs from uninfested host plant fitness 10

10. Infested holobiont plant fitness is greater than infested host plant fitness .... *Overcompensation;* go to 1a
10. Infested holobiont plant fitness is less than infested host plant fitness .... *Susceptibility;* go to 1b

1a. Uninfested holobiont fitness is greater than uninfested host plant fitness .... *Constitutive*
1a. Uninfested holobiont fitness is less than or equal to uninfested host plant fitness .... *Induced*

1b. Uninfested holobiont fitness is less than uninfested host plant fitness................ *Constitutive*
1b. Uninfested holobiont fitness is greater than or equal to uninfested host plant fitness….*Induced*
