# Supplementary material for: Root microbes can improve plant tolerance to insect damage: A systematic review and meta‐analysis
Source: Ecology. 2025 Jan 21;106(1):e4502. doi: 10.1002/ecy.4502 (PMC11750633; doi:10.1002/ecy.4502)
Supplement: Supplementary file 3 — Appendix S3. [file ECY-106-e4502-s001.pdf]

Root microbes can improve plant tolerance to insect damage: A systematic review and meta-analysis. Emily Tronson, Laramy Enders. *Ecology*.

Appendix S3: Supplemental Figures

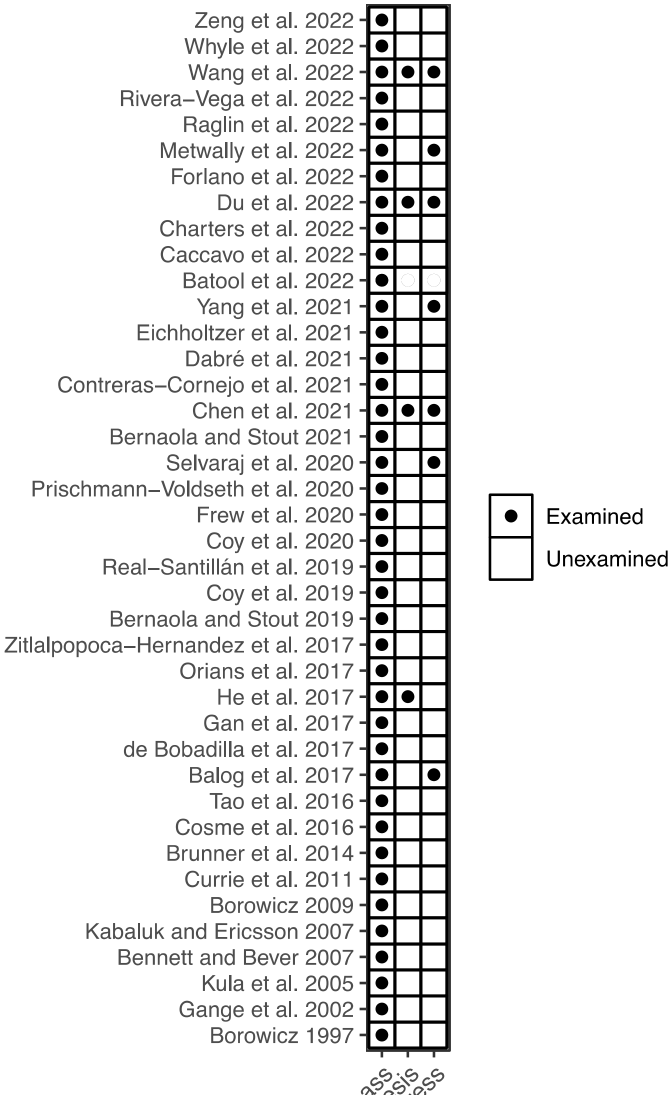

Appendix S3: Figure S1. Summary of the fitness measures used to estimate tolerance across studies included in this systematic review: photosynthesis, oxidative stress, and/or biomass under herbivory.

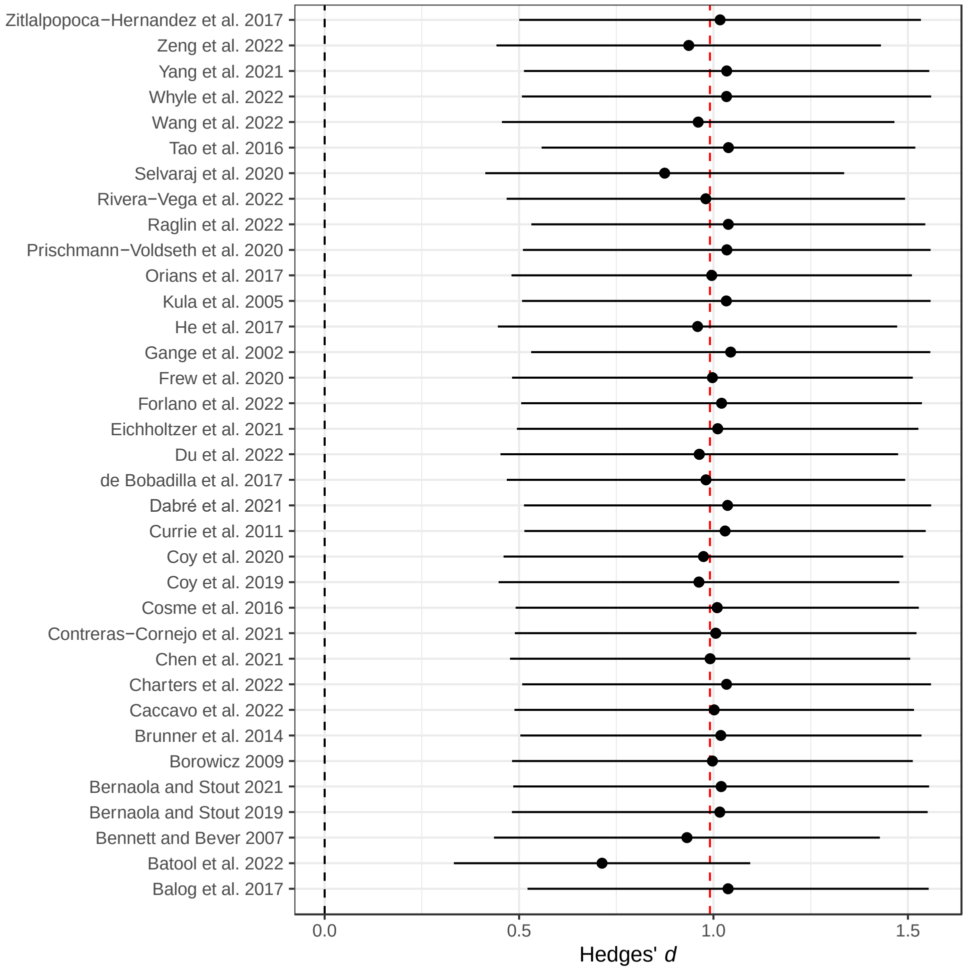

Appendix S3: Figure S2. Results of a leave-one-out analysis. We reran our main random effects model (effect sizes ~ Citation:Experiment:Observation), iteratively dropping each of the 40 papers included in the meta-analysis (y axis) from the dataset. The plotted Hedges' *d* values represent the grand mean effect size of this model without the citation provided on the y axis. The red vertical dashed line represents the grand mean effect size of this meta-analysis, while the black vertical dashed line represents 0. The red shaded rectangle represents the countries of the grand mean effect size's 95% confidence interval.
